# Reduction of MRSA Bacteremia LabID Events in a Large Community Tertiary Care Facility

**DOI:** 10.1017/ash.2025.363

**Published:** 2025-09-24

**Authors:** Kay Sams, S. Elizabeth Alvarez, Leslie Montalvo Gonzalez, Tracy Rummel, Mehrunnisa Khan, Vincent Hsu, Xiomara Hewitt, Tara Rhone

**Affiliations:** 1 Advent Health Tampa; 2 Adventhealth

## Abstract

**Background:** Methicillin-resistant Staphylococcus aureus (MRSA) bacteremia is a reportable hospital -acquired condition that can cause significant harm to hospitalized patients. Our facility, a 629 bed acute tertiary care hospital, continued to observe Healthcare Facility-Onset (HO) MRSA bacteremia rates above the corporate goal despite our housewide MRSA decolonization protocol consisting of daily chlorhexidine gluconate bathing (CHG) and an alcohol nasal antiseptic twice per day for all adult inpatients ≥ 18 years of age. This prompted us to conduct a gap analysis and evidence-based practice review to address our current MRSA decolonization practices. **Methods:** In January 2024, a revised MRSA decolonization protocol was implemented for our adult inpatient population consisting of the addition of nasal mupirocin twice per day for 5 days for all Intensive Care Unit (ICU) patients, MRSA nasal screening for high-risk patients, and implementation of contact precautions (gown, gloves) for patients identified or known with MRSA colonization and/or infection. The nasal alcohol antiseptic was removed from the revised protocol. Multidisciplinary education on the protocol changes (MRSA screening, isolation, and discontinuation of the nasal antiseptic) were disseminated to nursing, pharmacy, and the medical staff. Mupirocin was added to the standing ICU order set in the electronic medical record (EMR). Pre-intervention (February 2023 - January 2024) and Post-intervention (February 2024-October 2024) time periods were used to assess the impact on the rate of HO MRSA bacteremia and were obtained from The National Healthcare Safety Network (NHSN) standardized infection ratio (SIR) 2015 baseline with analysis using the NHSN statistics calculator. **Results:** Following implementation of the decolonization protocol, the MRSA bacteremia cumulative SIR decreased 91% from 1.077 to 0.096 which was statistically significant with a two-tailed p-value of 0.0021 (95% Confidence interval: -99.6, -49.5). The housewide MRSA bacteremia rate decreased from 1.05 infections per 10,000 patient days to 0.090 per 10,000 patient days which reflected a significant decrease (p: 0.0016). The ICU MRSA bacteremia rate also showed statistical significance with a decrease from 4.2 per 10,000 patient days to 0.00 per 10,000 patient days (p: 0.0477). **Conclusion:** Revising the MRSA decolonization protocol significantly decreased our MRSA bacteremia rates. This included re-implementation of contact precautions, screening high-risk patients that could be carriers of MRSA, and mupirocin decolonization for ICU patients. Facilities should consider evaluation of their MRSA decolonization, isolation, and screening practices if unable to decrease their HO MRSA bacteremia rates.

